# Supplementary material for: A set of multi-entry identification keys to African frugivorous flies (Diptera, Tephritidae)
Source: Zookeys. 2014 Jul 24;(428):97–108. doi: 10.3897/zookeys.428.7366 (PMC4143993; doi:10.3897/zookeys.428.7366)
Supplement: Supplementary material 6 — Key to Ceratitis [file zookeys-428-097-s006.zip › SF6_ZooKeys_key to Ceratitis/key/SF6_ZooKeys_key to Ceratitis/Media/Html/Ceratitis dumeti .htm]

Ceratitis (Ceratalaspis) dumeti Munro


***Ceratitis*** **(*Ceratalaspis*)
*dumeti* Munro**

*Ceratitis* (*Pardalaspis*) *dumeti*
Munro, 1933a: 35.

Body
length: 4.74 (4.15-5.10) mm; wing length: 4.29 (4.15-4.60) mm.

Male

Head.
Antenna yellow-orange. Third antennal segment twice as long as second segment.
Arista with short hairs over entire length. Frons with short scattered hairs
which are distinctly darker than frons; convex, in lateral view not distinctly
projecting forwards at antennal implant. Chaetotaxy normal for subgenus.

Thorax.
Postpronotum yellow-white, paler than centre mesonotum; unspotted. Ground
colour of mesonotum pale with yellow-orange tinge; mesonotum, sometimes
prescutellar spots weakly connected with dorsocentral setae, presutural spots
variable and occasionally median line present. Chaetotaxy normal for subgenus.
Scapular setae pale. One or two anepisternal bristles, lower bristle usually
smaller and less developed, occasionally palish colour. Scutellum white
basally, otherwise yellow with three black separate markings apically; basally
without dark spots, only slight darker colouration. Subscutellum three black
spots at same position as apical scutellar spots, widely separated.

Legs
yellow; setation typical for subgenus, mainly pale especially on femora.
Ventral, posterior and posterodorsal rows on fore femur pale.

Wing
bands with extensively yellow markings. Banding, marginal band continuous;
cubital band free, medial band absent; crossvein r-m before middle of discal
cell. Crossvein dm-cu posteriorly more inwards than anteriorly.

Abdomen.
Yellow. Setation and banding typical for subgenus.

Female

As
male except for the following characters: Antenna more yellow. Subscutellar
markings narrowly separated (as in other *Ceratalaspis* spp.). Mesonotum
with more outspoken orange tinge. Oviscape longer than abdominal terga 3-6
combined, or at least as long as terga.

(Description
after De Meyer, 1998)
